# P2X7 receptor-mediated PARP1 activity regulates astroglial death in the rat hippocampus following status epilepticus

**DOI:** 10.3389/fncel.2015.00352

**Published:** 2015-09-04

**Authors:** Ji Yang Kim, Ah-Reum Ko, Ji-Eun Kim

**Affiliations:** Department of Anatomy and Neurobiology, Institute of Epilepsy Research, College of Medicine, Hallym UniversityOkcheon-dong, Chuncheon, South Korea

**Keywords:** astrocyte, P2X7 receptor, PAR, PARP1, status epilepticus

## Abstract

Poly(ADP-ribose) polymerase-1 (PARP1) plays a regulatory role in apoptosis, necrosis, and other cellular processes after injury. Recently, we revealed that PARP1 regulates the differential neuronal/astroglial responses to pilocarpine-induced status epilepticus (SE) in the distinct brain regions. In addition, P2X7 receptor (P2X7R), an ATP-gated ion channel, activation accelerates astroglial apoptosis, while it attenuates clasmatodendrosis (lysosome-derived autophagic astroglial death). Therefore, we investigated whether P2X7R regulates regional specific astroglial PARP1 expression/activation in response to SE. In the present study, P2X7R activation exacerbates SE-induced astroglial apoptosis, while P2X7R inhibition attenuates it accompanied by increasing PARP1 activity in the molecular layer of the dentate gyrus following SE. In the CA1 region, however, P2X7R inhibition deteriorates SE-induced clasmatodendrosis via PARP1 activation following SE. Taken together, our findings suggest that P2X7R function may affect SE-induced astroglial death by regulating PARP1 activation/expression in regional-specific manner. Therefore, the selective modulation of P2X7R-mediated PARP1 functions may be a considerable strategy for controls in various types of cell deaths.

## Introduction

It has been reported that astroglial death/damage occurs following epilepsy, brain ischemia and Alzheimer’s diseases (Liu et al., 1999; Kang et al., 2006; Olabarria et al., 2010). In particular, status epilepticus (SE) induces the regional specific astroglial death (Kang et al., 2006; Kim et al., 2008; Kim and Kang, 2011; Ryu et al., 2011a,b). Briefly, caspase-independent apoptotic astroglial death is observed in the molecular layer of the dentate gyrus (Kang et al., 2006; Kim et al., 2010). In contrast, astroglial death in the CA1 region shows extensive swelling and vacuolization of cell bodies, and/or disintegrated and beaded processes in the CA1 region (Kim et al., 2008; Kim and Kang, 2011). This irreversible astroglial degeneration is first reported by Alzheimer in 1910, termed “clasmatodendrosis” by Cajal (Penfield, 1928), and is relevant to lysosome-derived autophagy (Ryu et al., 2011a,b).

P2X7 receptor (P2X7R), an ATP-gated ion channel, regulates the release of gliotransmitters and cytokines/chemokines from neuroglia (Le Feuvre et al., 2003; Parvathenani et al., 2003; Melani et al., 2005; McLarnon et al., 2006). Interestingly, P2X7R activation accelerates astroglial apoptosis, while it attenuates clasmatodendrosis (Kim et al., 2011). However, P2X7R function does not affect astroglial loss in the piriform cortex (PC, Kim et al., 2011). Therefore, it is likely that at least in the hippocampus P2X7R may be one of the fundamental effectors for regional specific astroglial loss induced by SE.

Poly(ADP-ribose) polymerase-1 (PARP1) repairs DNA damage following various injuries. Therefore, PARP1 plays an important role in anti-apoptotic events. Indeed, full-length PARP1 is cleaved by the apoptotic proteases (caspase-3 and -7) into p85 and p25 fragments during apoptosis (Kaufmann et al., 1993; Lazebnik et al., 1994). Furthermore, the down-regulation of PARP1 expression (PARP1 degradation without cleavage into apoptotic fragments) is mediated by caspase-independent ubiquitylation that plays a regulatory role in apoptosis, necrosis and other PARP1-regulated cellular processes (Guillouf et al., 2000; Wang et al., 2008; Matsushima et al., 2011; Nagai et al., 2012). However, PARP1 over-activation also causes necrotic cell death induced by NAD^+^-depleted energy failure, since PARP1 utilizes NAD^+^ to form PAR during DNA repairs (Berger, 1985; Zhang et al., 1994; Szabó and Dawson, 1998; Ha and Snyder, 1999; Virág and Szabó, 2002; Ying et al., 2002). Therefore, it is likely that the distinct profiles of PARP1 (activation, cleavage or degradation) may involve the differential cellular responses to harmful stimuli. Recently, we have reported that PARP1 degradation is observed in astrocytes within the molecular layer of the dentate gyrus, while PARP1 induction is detected in CA1–3 reactive astrocytes. Furthermore, PARP1 inhibitors deteriorate the SE-induced astroglial death in the molecular layer of the dentate gyrus independently of hemodynamics (Kim et al., 2014). Therefore, PARP1 activation/degradation may distinctly involve regional-specific astroglial death in response to SE, although the mechanism has not been fully understood. With respect to the role of P2X7R in SE-induced astroglial degeneration, it is plausible that P2X7R may involve astroglial PARP1 degradation/activation in response to SE. In order to address this hypothesis, we investigated the role of P2X7R in PARP1-mediated regional specific astroglial death induced by SE.

## Materials and Methods

### Experimental Animals and Chemicals

This study utilized the progeny of Sprague-Dawley (SD) rats (male, 9–11 weeks old) obtained from Experimental Animal Center, Hallym University, Chuncheon, South Korea. The animals were provided with a commercial diet and water *ad libitum* under controlled temperature, humidity and lighting conditions (22 ± 2°C, 55 ± 5% and a 12:12 light/dark cycle with lights). Animal protocols were approved by the Institutional Animal Care and Use Committee of Hallym University (No. 2013-107). Procedures involving animals and their care were conducted in accord with our institutional guidelines that comply with NIH Guide for the Care and Use of Laboratory Animals (NIH Publications No. 80-23, 1996). The number of animals used and their suffering were minimized in all cases. All reagents were obtained from Sigma-Aldrich (St. Louis, MO, USA), except as noted.

### Intracerebroventricular Drug Infusion

Rats were divided into four groups: vehicle (saline) treated, 2′,3′-O-(4-benzoylbenzoyl)-adenosine 5′-triphosphate (BzATP, P2X7R agonist, 5 mM, Sigma) treated, adenosine 5′-triphosphate-2′,3′-dialdehyde (OxATP, P2X7R antagonist, 5 mM, Sigma) treated and A740003 (P2X7R antagonist, 5 mM, Sigma) treated groups. The dosage of each compound was determined as the highest dose that did not affect seizure threshold in previous study (Kim et al., 2011). Animals were anesthetized using isoflurane and placed in a stereotaxic frame. For the osmotic pump implantation, holes were drilled through the skull for introducing a brain infusion kit 1 (Alzet, USA) into the right lateral ventricle (1 mm posterior; 1.5 mm lateral; −3.5 mm depth; flat skull position with bregma as reference), according to the atlas of Paxinos and Watson (1997). The infusion kit was sealed with dental cement and connected to an osmotic pump (1007D, Alzet, USA). The pump was placed in a subcutaneous pocket in the dorsal region. Animals received 0.5 μl/h of vehicle or compound for 1 week (Siuciak et al., 1996; Pencea et al., 2001).

### Seizure Induction

One week after surgery, rats were treated with pilocarpine (380 mg/kg, i.p.) 20 min after injection of methyl scopolamine (5 mg/kg, i.p.). Approximately 80% of pilocarpine treated rats showed acute behavioral features of SE (including akinesia, facial automatisms, limbic seizures consisting of forelimb clonus with rearing, salivation, masticatory jaw movements, and falling). Diazepam (10 mg/kg, i.p.) was administered 2 h after onset of SE and repeated, as needed. At designated time courses (3 days and 4 weeks after SE; *n* = 15, respectively), animals were used for immunohistochemistry. Non-experienced SE rats (showed only acute seizure behaviors during 10–30 min, *n* = 11) and age-matched normal rats were used as controls (*n* = 8).

### Tissue Processing

Animals were perfused transcardially with phosphate-buffered saline (PBS) followed by 4% paraformaldehyde in 0.1 M phosphate buffer (PB, pH 7.4) under urethane anesthesia (1.5 g/kg, i.p.). The brains were removed, and postfixed in the same fixative for 4 h. The brain tissues were cryoprotected by infiltration with 30% sucrose overnight. Thereafter, the entire hippocampus was frozen and sectioned with a cryostat at 30 μm and consecutive sections were contained in six-well plates containing PBS. For stereological study, every sixth section in the series throughout the entire hippocampus was used in some animals.

### Immunofluorescence Staining

To identify the morphological changes induced by SE in the same hippocampal tissue, double immunofluorescence staining was performed. Brain tissues were incubated with a mixture of mouse anti-GFAP IgG (diluted 1:100; Millipore, Bedford, MA, USA)/rabbit anti-PARP1 IgG (diluted 1:100; Abnova), rabbit anti-GFAP IgG (diluted 1:200; Promega, Madison, WI, USA)/mouse anti-PAR IgG (diluted 1:100; Trevigen, Gaithersburg, MD, USA) or mouse anti-GFAP IgG/rabbit anti-lysosomal-associated membrane protein-1 (LAMP1) IgG (diluted 1:100; Abcam, USA) overnight at room temperature. After washing three times for 10 min with PBS, sections were also incubated in a mixture of FITC- and Cy3-conjugated secondary antisera (Amersham, USA, 1:200) for 1 h at room temperature. Sections were mounted in Vectashield mounting media with/without DAPI (Vector, Burlingame, CA, USA). Images were captured using an AxiocamHRc camera and Axio Vision 3.1 software or Bio-Rad MRC 1024 Confocal Microscope System (Bio-Rad Laboratories, 2000 Alfred Nobel Drive, Hercules, CA 94547, USA). Figures were mounted with Adobe PhotoShop 7.0 (San Jose, CA, USA). Manipulation of the images was restricted to threshold and brightness adjustments to the whole image.

### Cell Counts

For quantification of immunohistochemical data, Cells in 2–4 regions (1 × 10^5^μm^2^) from each section were counted on 20 × images. Results are presented as means ± SEM of 15–24 regions from five animals. For quantification of PARP1, PAR or LAMP1 in astrocytes, immunofluorescent images (10 sections/rat) were captured (500 × 500 μm). Images were sampled from at least five different points within each section. Thereafter, the number of GFAP positive cells showing PARP1, PAR or LAMP1 induction was actually counted within the sampled images. All immunoreactive cells were counted regardless the intensity of labeling. Cell counts were performed by two different investigators who were blind to the classification of tissues. All data obtained from the quantitative measurements were analyzed using one-way ANOVA to determine statistical significance. Bonferroni’s test was used for *post hoc* comparisons. A *p*-value below 0.05 was considered statistically significant (Kim et al., 2008, 2009).

## Results

### P2X7R does not Affect Neuronal PARP1 Expression in Response to SE

**Figure 1:**
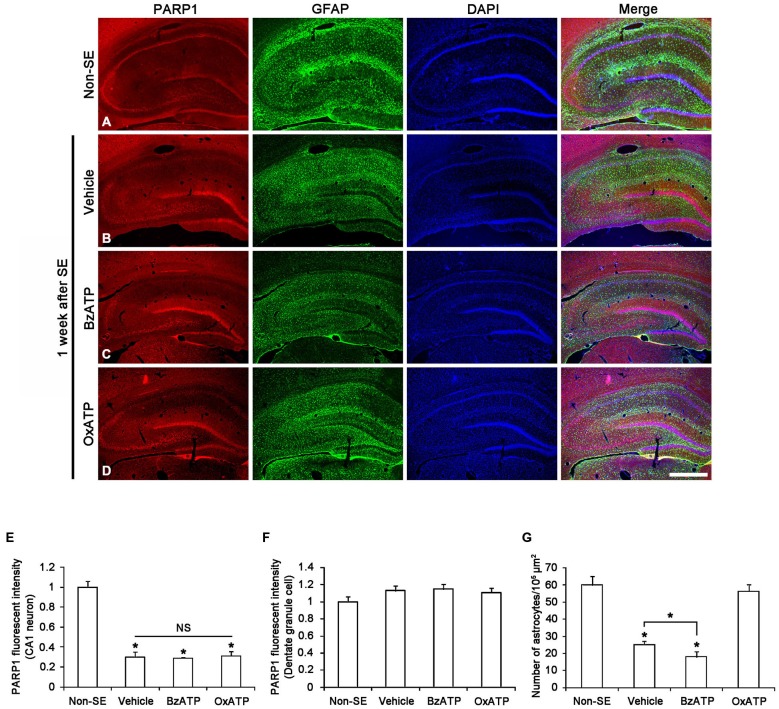
**The effects of P2X7R agonist and antagonist on PARP1 expression in the hippocampus 1 week after status epilepticus (SE).** As compared to non-SE animals, PARP1 expression is decreased in CA1 neurons, but not dentate granule cells **(A,B)**. Neither BzATP nor OxATP affects reduction in PARP1 expression in the CA1 neurons following SE **(C,D)**. Furthermore, SE reduces the number of astrocytes in the molecular layer of the dentate gyrus. BzATP deteriorates SE-induced astroglial loss in this region, while OxATP prevents it **(A–D)**. Bar = 300 μm. **(E,F)** Quantification of PARP1 intensity in the CA1 **(E)** and dentate granule cells **(F)** and the number of astrocytes in the molecular layer of the dentate gyrus **(G)** following SE (means ± s.d., *n* = 5, respectively). *p < 0.05 vs. non-SE animals (one-way ANOVA test).

Recently, we have reported that PARP1 activation/expression shows regional- and cellular specific responses to seizure in a hemodynamic-independent manner (Kim et al., 2014). Thus, we investigated whether P2X7R activity affects the SE-induced changes of PARP1 expression in neurons. Figure 1A shows that PARP1 expression was evenly observed in CA1–3 neurons as well as dentate granule cells in non-SE animals. In non-SE animals, P2X7R agonist and antagonist infusion did not affect neuronal PARP1 expression and neuronal viability in the hippocampus (data not shown). Consistent with our previous study (Kim et al., 2014), PARP1 expression was down-regulated in CA1 neurons 1 week after SE (*p* < 0.05 vs. non-SE animals; Figures 1B,E). However, PARP1 expression was unaltered in dentate granule cells in this time window (Figure 1F). Both BzATP and OxATP infusion did not affect neuronal PARP1 expression in the hippocampus 1 week after SE (Figures 1C–F). These findings indicate that P2X7R may not be involved in PARP1-mediated neuronal death induced by SE.

### P2X7R Antagonists Prevent PARP1-Medaited Astroglial Death in the Dentate Gyrus Following SE

To confirm the role of P2X7R in astroglial death in the dentate gyrus, we applied P2X7R agonist (BzATP) and antagonist (OxATP or A7400036) infusion prior to SE induction. Consistent with our previous study (Kim et al., 2011), P2X7R agonist and antagonist infusion did not lead to astroglial apoptosis in the hippocampus of non-SE animal (data not shown). One week after SE, typical reactive astrogliosis was observed in the stratum radiatum of the CA1 region (Figures 1B,C). However, massive astroglial loss was detected within the molecular layer of the dentate gyrus in this time point. In vehicle-infused animals, the number of astroglial cells was reduced to 42% of that in non-SE animals (*p* < 0.05 vs. non-SE animals; Figures 1B,G). In BzATP-infused animals, the number of astroglial cells was reduced to 30% of that in non-SE induced animals (*p* < 0.05 vs. vehicle-treated animals; Figures 1C,G). In OxATP-infused animals, the number of astroglial cells was 94% of that in non-SE animals (Figures 1D,G). The effect of A740003 on astroglial responses to SE was similar to that of OxATP (data not shown). Consistent with our previous studies (Kim et al., 2011), these findings indicate that P2X7R activation may exacerbate SE-induced astroglial death, while P2X7R inhibition may attenuate it in the molecular layer of the dentate gyrus following SE.

**Figure 2:**
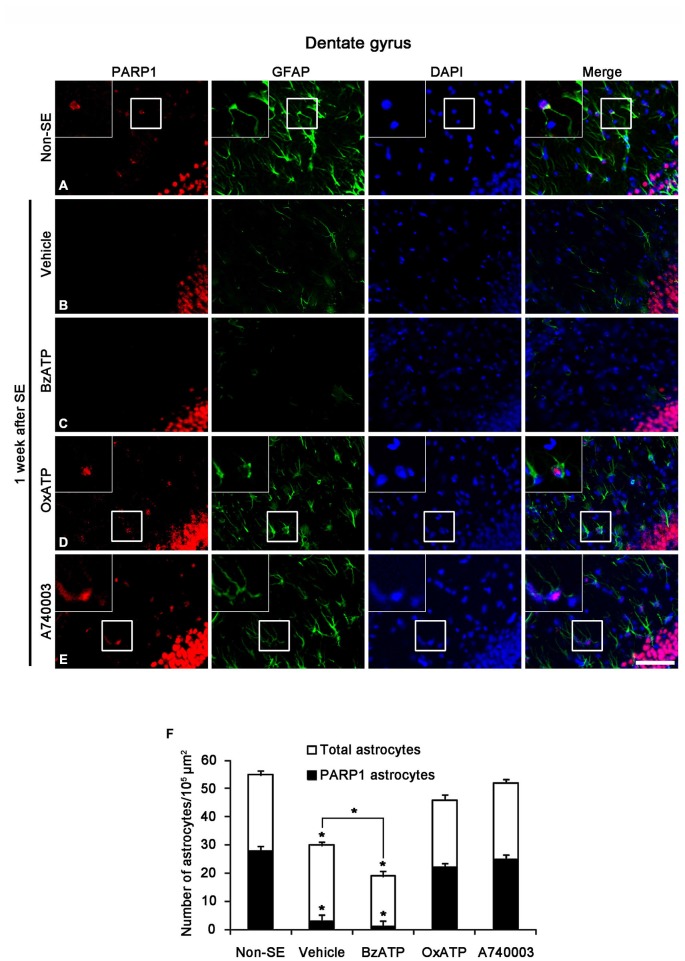
**The effects of P2X7R agonist and antagonist on PARP1 expression in astrocytes within the molecular layer of the dentate gyrus 1 week after SE.** As compared to non-SE animals **(A)**, vehicle-treated animals show the astroglial loss in the molecular layer of the dentate gyrus accompanied by reduction of PARP1 expression **(B).** BzATP deteriorates astroglial death and astroglial PARP1 degradation following SE **(C).** Both OxATP and A740003 effectively prevent astroglial death with the preservation of astroglial PARP1 expression **(D,E)**. Insertions (in **A,D,E**) are high magnification of rectangles. Bar = 50 and 25 (insertion) μm. **(F)** The fraction of PARP1 positive astrocytes in the total number of astrocytes within the molecular layer of the dentate gyrus (means ± s.d., *n* = 5, respectively). *p < 0.05 vs. non-SE animals (one-way ANOVA test).

SE-induced down-regulation of PARP1 expression results in astroglial death within the molecular layer of the dentate gyrus (Kim et al., 2014). Therefore, we investigated whether P2X7R is involved in PARP1-mediated astroglial death in this region following SE. In non-SE animals, 51% of astrocytes showed PARP1 expression in the molecular layer of the dentate gyrus (Figures 2A,F). P2X7R agonist and antagonist infusion did not affect astroglial PARP1 expression in the hippocampus of non-SE animal (data not shown). One week after SE, 10% astrocytes showed PARP1 expression (*p* < 0.05 vs. non-SE animals; Figures 2B,F). BzATP did not affect SE-induced astroglial PARP1 expression as compared to vehicle (*p* < 0.05 vs. non-SE animals; Figures 2C,F). In contrast to BzATP, both OxATP and A740003 effectively prevented the down-regulation of astroglial PARP1 expression as well as astroglial loss induced by SE (*p* < 0.05 vs. vehicle; Figures 2D–F).

**Figure 3:**
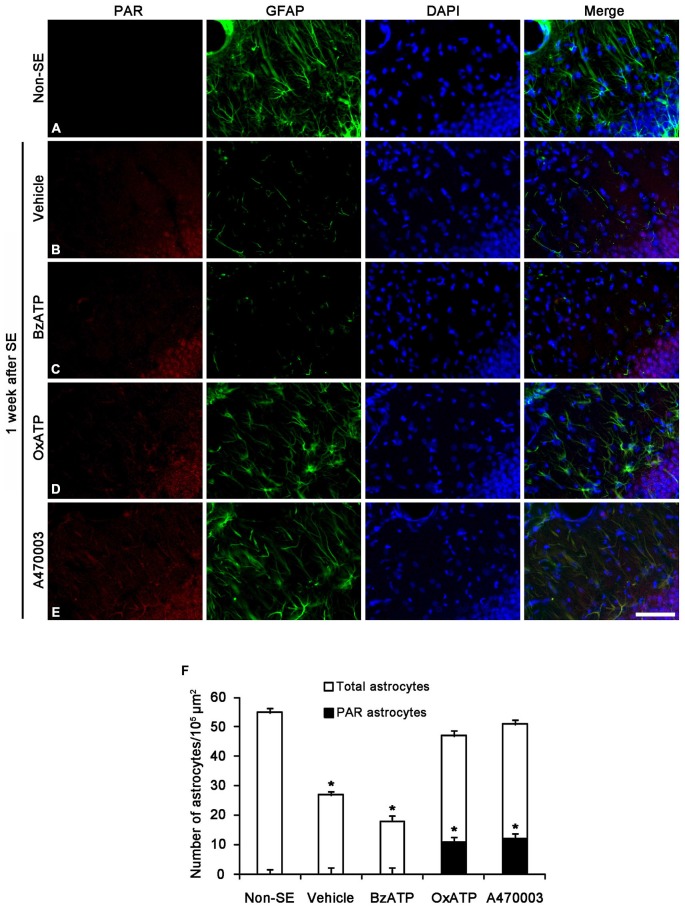
**The effects of P2X7R agonist and antagonist on PARP1 activity in the molecular layer of the dentate gyrus 1 week after SE.** PAR synthesis is detected only in dentate granule cells **(A,B)**. BzATP does not affect astroglial PAR synthesis induced by SE **(C).** Both OxATP and A740003 increase astroglial PAR expression following SE **(D,E)**. Bar = 50 μm. **(F)** The fraction of PAR positive astrocytes in the total number of astrocytes within the molecular layer of the dentate gyrus (means ± s.d., *n* = 5, respectively). *p < 0.05 vs. non-SE animals (one-way ANOVA test).

Since PAR is a metabolite of PARP1 (Zhang et al., 1994; Virág and Szabó, 2002; Ying et al., 2002), we also performed Immunohistochemistry for PAR to investigate astroglial PARP1 activity. Consistent with our previous study (Kim et al., 2014), PAR immunoreactivity was detected only in the dentate granule cells 1 week after SE (Figures 3A,B). Although BzATP did not affect astroglial PAR synthesis (Figure 3C), both OxATP and A740003 increased astroglial PAR levels in this time point (*p* < 0.05 vs. vehicle; Figures 3D–F). Therefore, 23- and 24% of astrocytes showed PAR immunoreactivity in OxATP and A740003-treated animals, respectively. Neither OxATP nor A740003 affected neuronal PAR level in this time window (Figures 3D–F). These findings indicate that P2X7R inhibition may prevent astroglial loss by increasing PARP1 activity in the molecular layer of the dentate gyrus following SE.

### P2X7R Inhibitors Aggravate Autophagic Astroglial Death with PARP1 Activation in the CA1 Region

**Figure 4:**
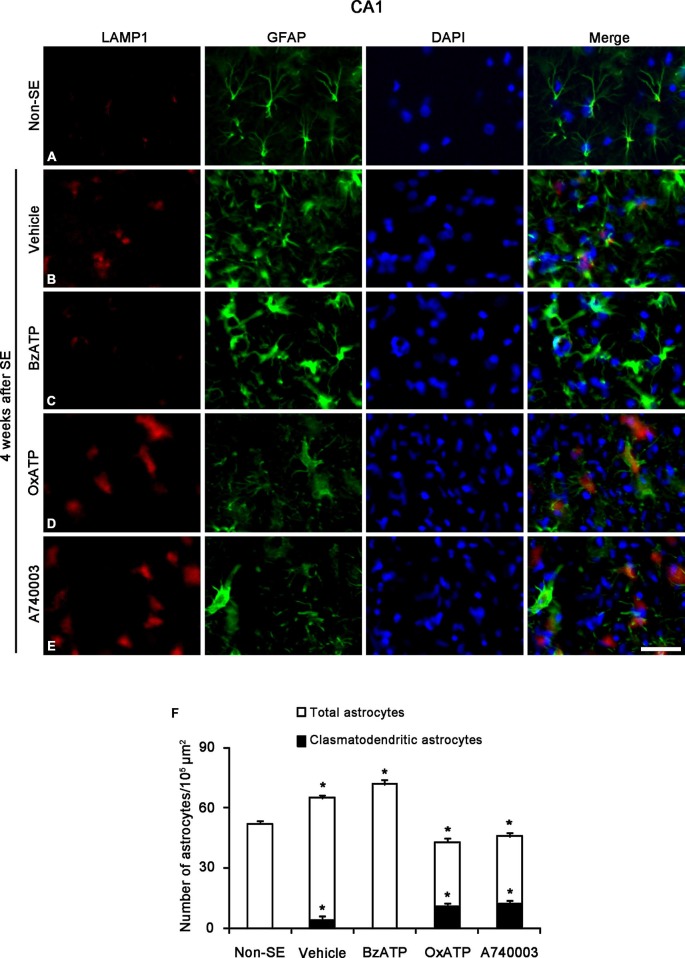
**The effects of P2X7R agonist and antagonist on clasmatodendrosis in the stratum radiatum of the CA1 region 4 weeks after SE.** SE induces lysosomal-associated membrane protein-1 (LAMP1) positive clasmatodendrosis in the CA1 region **(A,B)**. BzATP effectively reduces the number of LAMP1 positive clasmatodendritic astrocytes **(C).** Both OxATP and A490003 aggravate LAMP1 positive clasmatodendrosis **(D,E)**. Bar = 25 μm. **(F)** The fraction of LAMP1 positive astrocytes in the total number of astrocytes within the CA1 region (means ± s.d., *n* = 5, respectively). *p < 0.05 vs. non-SE animals (one-way ANOVA test).

Clasmatodendrosis is characterized by round-shaped edematous cell body, short blunt processes, vacuolization and GFAP tangles in the cytoplasm and nuclear dissolution. In addition, vacuoles in clasmatodendritic astrocytes showed LAMP1 (a marker for lysosome) immunoreactivity, not GM130 (a maker for Golgi apparatus) or EEA-1 (a marker for endosome) immunoreactivity. Therefore, clasmatodendrosis is the slow astroglial death induced by lysosome-derived autophagy (Ryu et al., 2011a,b). Unlike apoptotic astroglial death in the molecular layer of the dentate gyrus, however, the role of PARP1 in clasmatodendrosis is unknown. Thus, we investigated whether P2X7R-mediated PARP1 activation/expression is involved in this autophagic astroglial death, since P2X7R inhibition accelerates clasmatodendrosis in the CA1 region (Kim et al., 2011). Consistent with our previous study (Kim et al., 2011), P2X7R agonist and antagonist infusion did not result in clasmatodendrosis in the CA1 region of non-SE animal (data not shown). Four weeks after SE, 6.1% of astrocytes showed typical clasmatodendrosis (loss of distal process and vacuolization in cell body) containing strong LAMP1 expression in the stratum radiatum of the CA1 region of vehicle-treated animals (*p* < 0.05 vs. non-SE animals; Figures 4A,B,F). While BzATP inhibited clasmatodendrosis (*p* < 0.05 vs. vehicle; Figures 4C,F), OxATP and A740003 increased the number of clasmatodendritic astrocytes in total astrocytes (*p* < 0.05 vs. vehicle; Figures 4D–F). In addition, both P2X7R antagonists significantly reduced the total number of astrocytes in the CA1 region as compared to vehicle (*p* < 0.05 vs. vehicle; Figure 4F).

**Figure 5:**
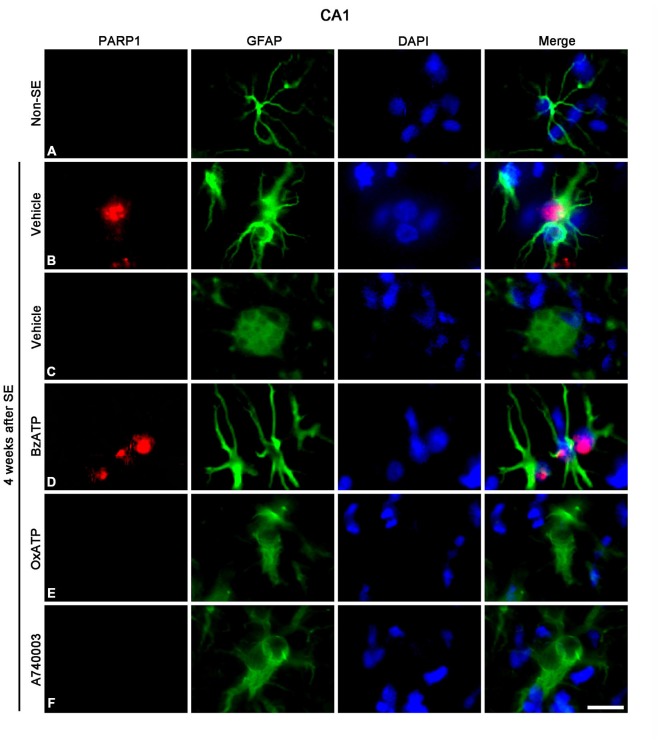
**The effects of P2X7R agonist and antagonist on astroglial PARP1 expression in the stratum radiatum of the CA1 region 4 weeks after SE.** PARP1 expression is rarely detected in astrocytes of non-SE animals **(A).** Following SE, PARP1 expression is observed in reactive astrocytes **(B)**, not in clasmatodendritic astrocytes **(C).** BzATP increases PARP1 expression in reactive astrocytes **(D).** Both OxATP and A740003 cannot induce PARP1 expression in clasmatodendritic astrocytes **(E,F)**. Bar = 12.5 μm.

**Figure 6:**
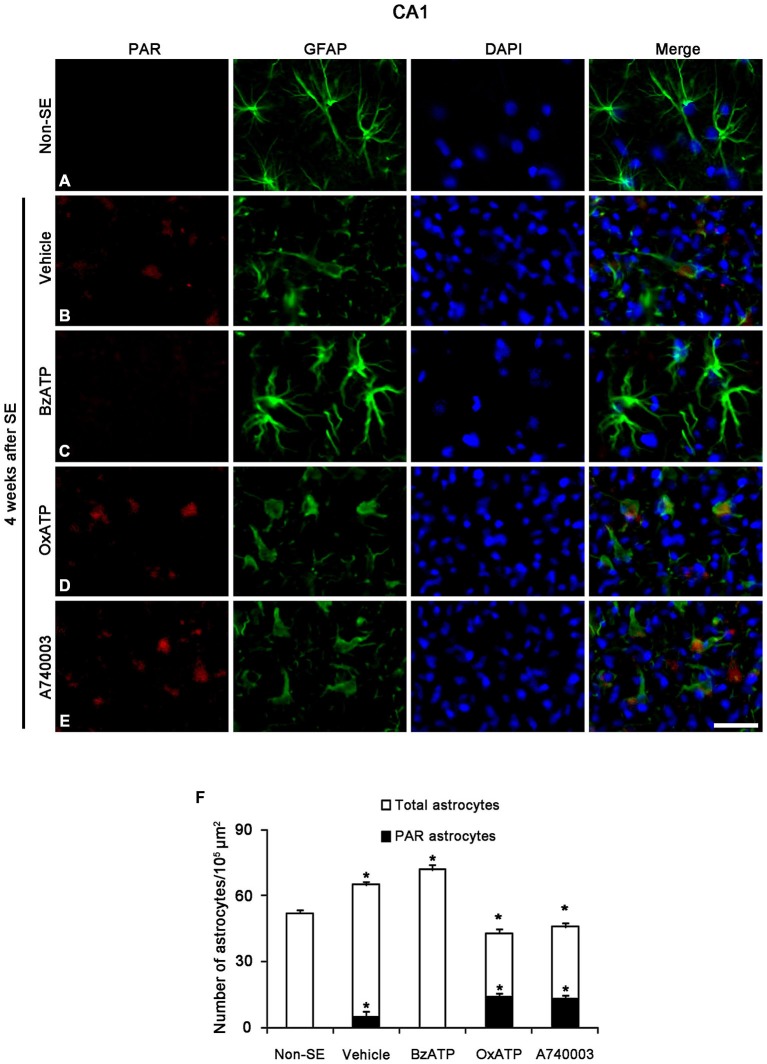
**The effects of P2X7R agonist and antagonist on astroglial PARP1 activity in the stratum radiatum of the CA1 region 4 weeks after SE.** PAR synthesis is rarely detected in astrocytes of non-SE animals **(A).** Following SE, PAR synthesis is observed in clasmatodendritic astrocytes **(B).** BzATP inhibits PAR synthesis in astrocytes. Both OxATP and A740003 increase the number of PAR positive astrocytes **(C–E)**. Bar = 25 μm. **(F)** The fraction of PAR positive astrocytes in the total number of astrocytes within the CA1 region (means ± s.d., *n* = 5, respectively). *p < 0.05 vs. non-SE animals (one-way ANOVA test).

**Figure 7:**
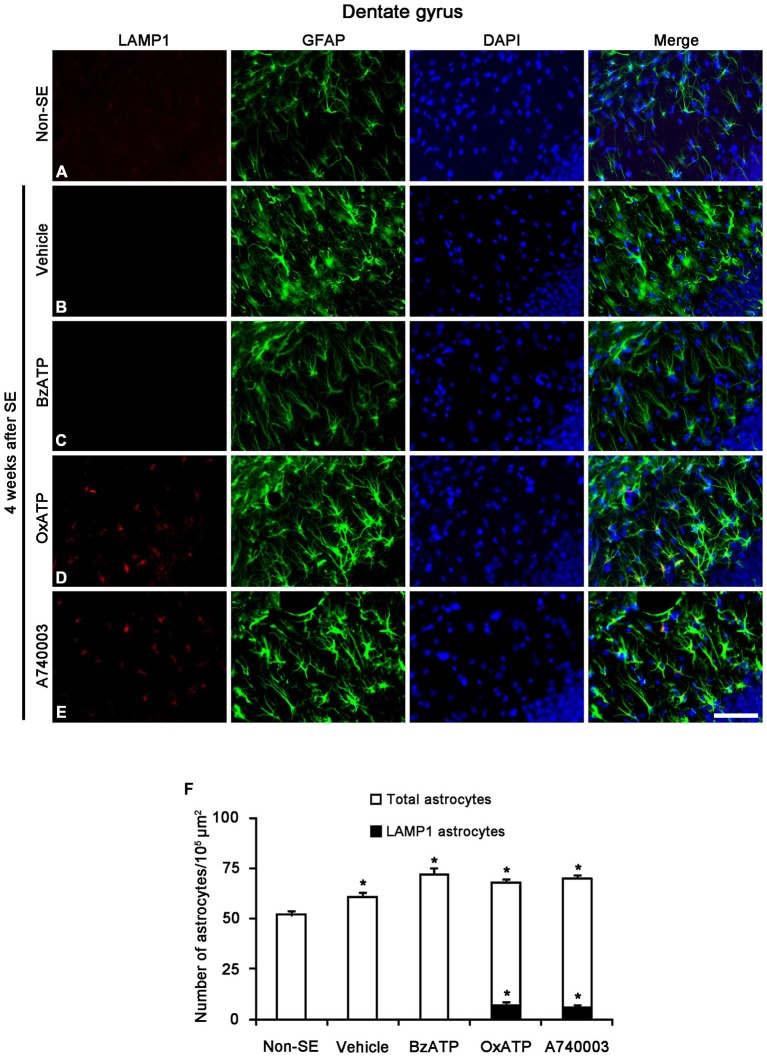
**The effects of P2X7R agonist and antagonist on LAMP1 expression in reactive astrocytes within the molecular layer of the dentate gyrus 4 weeks after SE.** In non-SE animals, LAMP1 positive astrocytes are not observed in the molecular layer **(A).** SE induces astroglial LAMP1 expression **(B)**, and BzATP does not affect it **(C).** However, both OxATP and A740003 increase the number of LAMP1 positive astrocytes in the molecular layer of the dentate gyrus **(D,E)**. Bar = 50 μm. **(F)** The fraction of LAMP1 positive astrocytes in the total number of astrocytes within the molecular layer of the dentate gyrus (means ± s.d., *n* = 5, respectively). *p < 0.05 vs. non-SE animals (one-way ANOVA test).

**Figure 8:**
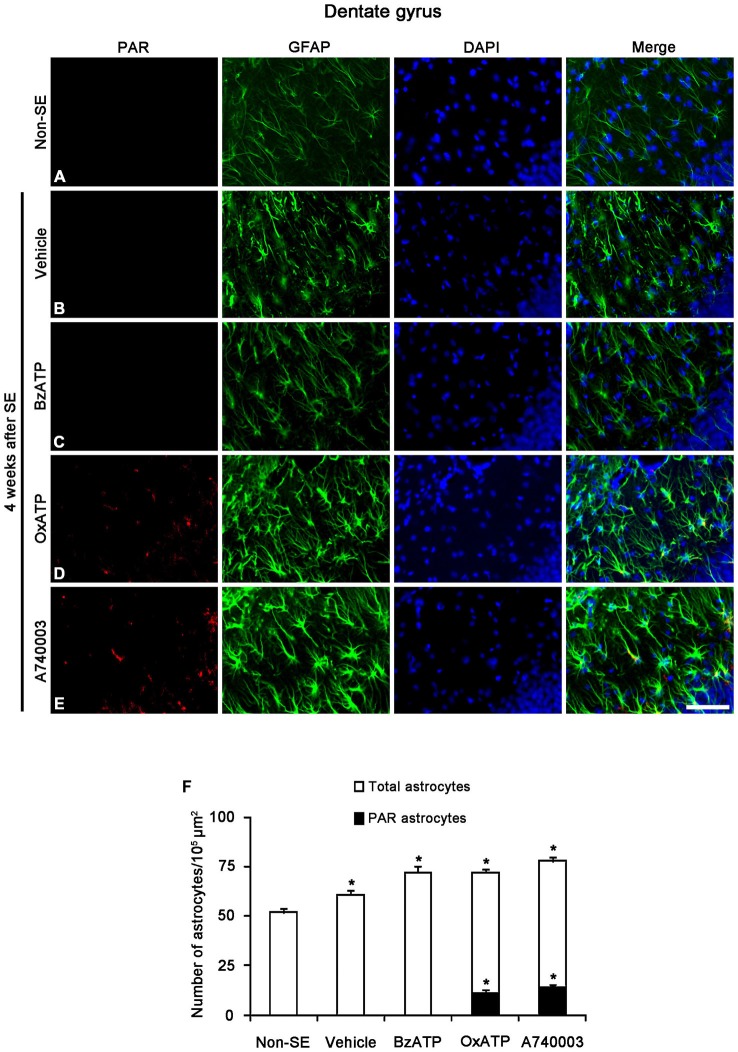
**The effects of P2X7R agonist and antagonist on PARP1 activity in reactive astrocytes within the molecular layer of the dentate gyrus 4 weeks after SE.** PAR-positive astrocytes are not observed in the molecular layer within the dentate gyrus of non-SE **(A)**, vehicle-treated **(B)** and BzATP-treated **(C)** animals. Both OxATP and A740003 increase the number of PAR positive astrocytes in the molecular layer of the dentate gyrus **(D,E)**. Bar = 50 μm. **(F)** The fraction of PAR positive astrocytes in the total number of astrocytes within the molecular layer of the dentate gyrus (means ± s.d., *n* = 5, respectively). *p < 0.05 vs. non-SE animals (one-way ANOVA test).

Next, we investigated whether P2X7R regulates astroglial PARP1 expression/activity. PARP1 expression was obviously detected in the nuclei of reactive astrocytes, but not in the nuclei of clasmatodendritic astrocytes (Figures 5A–C). BzATP effectively prevented clasmatodendrosis accompanied by preservation of the nuclear PARP1 expression, although P2X7R antagonists did not (Figures 5D–F). As compared to naïve astrocytes in non-SE animals, clasmatodendritic astrocytes showed PAR immunoreactivity (Figures 6A,B,F). BzATP effectively prevented astroglial PAR synthesis induced by SE (*p* < 0.05 vs. vehicle; Figures 6C,F). In contrast, OxATP and A740003 increased the fraction of PAR-positive clasmatodendritic astrocytes in total astrocytes to 25.6 and 26.1% respectively (*p* < 0.05 vs. vehicle; Figures 6D–F). In the molecular layer of the dentate gyrus, OxATP and A740003 also increased the fraction of PAR- and LAMP1 positive astrocytes in total astrocytes 4 weeks after SE (*p* < 0.05 vs. vehicle; Figures 7, 8). However, both OxATP and A740003 did not induce clasmatodendrosis and astroglial loss in this region (Figures 7, 8). These findings indicate that P2X7R antagonism may increase astroglial PARP1 activity and autophagic process in the hippocampus following SE.

### PARP1 Inhibition Attenuates SE-Induced Clasmatodendrosis in the CA1 Region

**Figure 9:**
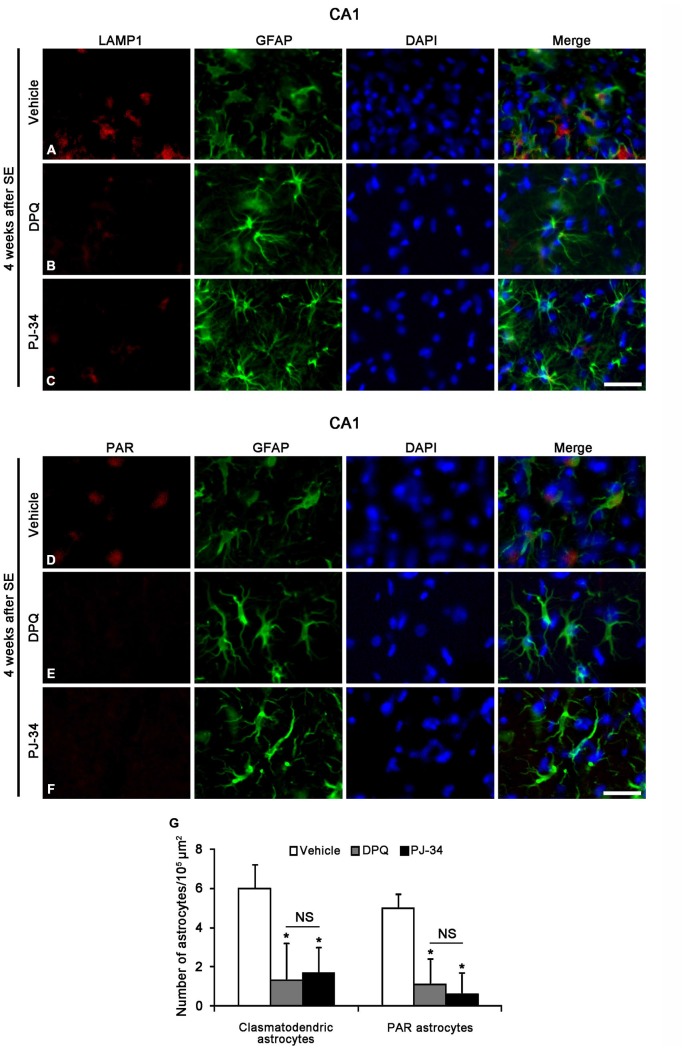
**The effects of PARP1 inhibitors (DPQ and PJ-34) on LAMP1 or PAR positive astroglial responses in the stratum radiatum of the CA1 region 4 weeks after SE.** Both PARP1 inhibitors reduce LAMP1 positive clasmatodendrosis **(A–C)** and PAR synthesis **(D–F)**, as compared to vehicle. **(G)** The numbers of clasmatodendritic and PAR-positive astrocytes in the stratum radiatum of the CA1 region (means ± s.d., *n* = 5, respectively). *p < 0.05 vs. vehicle-treated animals (one-way ANOVA test). Bar = 50 μm.

To confirm whether the enhancement of astroglial PARP1 activity leads to clasmatodendrosis in the CA1 region, we applied two PARP1 inhibitors (PJ-34 and DPQ) 2 week after SE. Both PJ-34 and DPQ effectively alleviated astroglial PAR synthesis and clasmatodendrosis in the CA1 region 4 weeks after SE (*p* < 0.05 vs. vehicle; Figure 9). These findings suggest that PARP1 activation may be involved in SE-induced astroglial autophagic process.

## Discussion

Astroglial death/dysfunction involves the pathophysiology of various neurological diseases (Briellmann et al., 2002; Vessal et al., 2005; Kang et al., 2006). In particular, SE results in the regional-specific astroglial damage characterized by a pattern of selective vulnerability (Schmidt-Kastner and Ingvar, 1994, 1996; Borges et al., 2003; Kang et al., 2006; Kim et al., 2010, 2011). However, the molecular events underlying the occurrence of SE-induced astroglial death has been unknown. In the present study, P2X7R agonist and its antagonists could not induce apoptotic astroglial loss in naïve astrocytes in non-SE animals. However, P2X7R inhibition prevented SE-induced astroglial loss accompanied by preservation of PARP1 expression and PAR synthesis in the molecular layer of the dentate gyrus, where acute astroglial apoptosis is observed 3–7 days after SE (Kim et al., 2010, 2011). Unlike neurons, this SE-induced astroglial apoptosis is mediated by apoptosis inducing factor (AIF). AIF is synthesized in the cytosol as a 67 kDa precursor protein and cleaved to produce a 57 kDa protein once transported into the mitochondria (Candé et al., 2002). Under pathological conditions, a soluble 57 kDa AIF protein translocates into the nucleus where it results in chromatin condensation and DNA fragmentation via caspase-independent manners (Joza et al., 2001; Candé et al., 2004; Cregan et al., 2004). PARP1 activation leads to cytosolic NAD^+^ depletion and mitochondrial release of AIF, and restoration of cytosolic NAD^+^ through P2X7R prevents release of AIF induced by PARP1 activation *in vitro* (Alano et al., 2004, 2010). Therefore, it would be likely that PARP1 activation might be involved in astroglial apoptosis in the dentate gyrus. However, PARP1 inhibitors cannot prevent SE-induced apoptotic astroglial death *in vivo* (Kim et al., 2014). In addition, the present data show that P2X7R antagonists prevented SE-induced astroglial loss in the dentate gyrus accompanied by the preservation of PARP1 expression, which may subsequently facilitate PARP1-mediated DNA repairs in astrocytes (Kaufmann et al., 1993; Lazebnik et al., 1994). Therefore, it is likely that SE-induced astroglial apoptosis may be PARP1 activity-independent processes.

In contrast to apoptotic event, clasmatodendrosis is relevant to severe intracellular acidosis that is relatively slow events (Kraig and Chesler, 1990; Hulse et al., 2001). This intracellular acidification induces clasmatodendrosis during hyperglycemic and energy failure (Friede and Van Houten, 1961; Kraig and Chesler, 1990; Hulse et al., 2001). In the present study, P2X7R agonist and its antagonists could not provoke clasmatodendrosis in naïve astrocytes in non-SE animals. However, we found that P2X7R antagonists promoted clasmatodendrosis, while P2X7R agonist prevented it 4 weeks after SE. Since P2X7R activation opens pannexin-1, which is one of hemichannel as a passage for NAD^+^ (Alano et al., 2004, 2010; Kim and Kang, 2011), the blockade of P2X7R may result in cytosolic NAD^+^ accumulation and prolonged PARP1 activation. However, excessive PARP1 activation leads to intracellular acidification with release of protons during hydrolysis of NAD^+^ to PAR (Affar el et al., 2002). Therefore, it is likely that PARP1 over-activation induced by SE in CA1 astrocytes may be an important factor inducing clasmatodendrosis through intracellular acidification. Indeed, the present study shows that both PARP1 inhibitors (PJ-34 and DPQ) effectively alleviated PAR synthesis and clasmatodendrosis in the CA1 region. Taken together, our findings indicate that blockade of P2X7R may accelerate astroglial autophagic processes by reinforcing PARP1-induced intracellular acidosis in astrocytes following SE.

In the present study, however, clasmatodendritic astrocytes showed PAR synthesis without nuclear PARP1 expression. Morphologically, clasmatodendritic astrocytes have edematous eosinophilic cytoplasm and large-sized vacuoles with pyknotic nucleus or without nucleus (Kim et al., 2009). This morphology of clasmatodendritic astrocytes is similar to that of ghost cells retaining their size and shape with digested nuclei, which are a consequence of coagulative necrosis (Kerr et al., 1972; Kim et al., 2009). Indeed, autophagy undergoes a change resembling coagulative necrosis (Searle et al., 1975; Kim et al., 2009; Ryu et al., 2011b). Therefore, absence of PARP1 expression may be due to the nuclear digestion during autophagic processes. Since ghost cells have a potentially much greater capacity to synthesize PAR (Benjamin and Gill, 1980), it is not surprising that clasmatodendritic astrocytes showed the amount of PAR synthesis without PARP1 expression.

Interestingly, the present data demonstrate that P2X7R antagonists also increased astroglial PARP1 activity and LAMP1 expression in the dentate gyrus 4 weeks after SE, although they did not induce clasmatodendrosis unlike CA1 astrocytes. These findings indicate that astrocytes in the dentate gyrus may be resistant to clasmatodendritic degeneration induced by P2X7R-mediated PARP1 activation, and also support the hypothesis that the susceptibility of astrocytes in response to SE is most likely due to the distinctive anatomical and physiological heterogeneity of astrocytes independent of hemodynamics.

In conclusion, the present study provides novel evidence that P2X7R function may affect SE-induced astroglial death by regulating PARP1 activation/expression in regional-specific manner. To the best of our knowledge, the present study demonstrates for the first time the relationship between P2X7R and PARP1 functions during SE-induced astroglial death. Therefore, we suggest that the selective modulation of P2X7R-mediated PARP1 functions may be a considerable strategy for controls in various types of cell deaths.

## Author Contributions

J-EK designed and supervised the project. J-EK designed and performed the experiments described in the manuscript with JYK and A-RK. JYK, A-RK and J-EK analyzed the data. J-EK wrote the manuscript.

## Conflict of Interest Statement

The authors declare that the research was conducted in the absence of any commercial or financial relationships that could be construed as a potential conflict of interest.
